# The prevalence of headache disorders among children and adolescents in Georgia: a cross-sectional schools-based study

**DOI:** 10.1186/s10194-025-02154-5

**Published:** 2025-12-04

**Authors:** Nana Nino Tatishvili, Zaza Katsarava, Teona Shatirishvili, Tamar Kipiani, Tinatin Giuashvili, Mariam Lomsianidze, Aleksandre Kotetishvili, Anano Kvernadze, Irakli Tugushi, Salome Maghlakelidze, Vera Nemsadze, Ana Bedoshvili, Tamar Bitskinashvili, Giorgi Sakvarelidze, Tayyar Şaşmaz, Deniz Erdal, Andreas Kattem Husøy, Derya Uludüz, Timothy J. Steiner

**Affiliations:** 1 David Tvildiani Medical University (DTMU), Tbilisi, Georgia; 2 Centre of Neurology, Geriatric Medicine and Early Rehabilitation, Evangelical Hospital, Unna, Germany; 3 Medical Faculty, University of Essen, Essen, Germany; 4 Neuroscience Department, M Iashvili Central Childrens Hospital, Tbilisi, Georgia; 5 Department of Public Health, Mersin University School of Medicine, Mersin, Turkey; 6 Department of Neuromedicine and Movement Science, Norwegian Centre for Headache Research (NorHead), Norwegian University of Science and Technology, Trondheim, Norway; 7 Department of Neurology and Clinical Neurophysiology, St. Olav University Hospital, Trondheim, Norway; 8 Neurology Department, Cerrahpaşa School of Medicine, Istanbul University, Istanbul, Turkey; 9 Department of Neurology, University of Copenhagen, Copenhagen, Denmark; 10 Division of Brain Sciences, Imperial College London, London, UK

**Keywords:** Headache disorders, Migraine, Tension-type headache, Medication-overuse headache, Undifferentiated headache, Epidemiology, Prevalence, Children, Adolescents, Cross-sectional study, Schools-based survey, Georgia, European region, Global Campaign against Headache

## Abstract

**Background:**

The Global Campaign against Headache is undertaking a worldwide programme of cross-sectional schools-based studies of headache prevalence and attributed burden among children (6–11 years) and adolescents (12–17). The purpose is to expand the still sparse knowledge of headache among these age groups, using a data-acquisition technique that cluster-samples the world. This survey in Georgia was the fourth in the European Region, following similar studies in Turkey, Austria and Lithuania, and others in African, Eastern Mediterranean, South-East Asia and Western Pacific Regions.

**Methods:**

We used the Global Campaign’s standardized protocol, selecting schools representative of the country. The child and adolescent versions of the structured HARDSHIP questionnaire, translated into Georgian language, were completed by pupils under supervision in class. Headache diagnoses followed ICHD-3, with the exception of undifferentiated headache (UdH), which we defined initially as mild and subsequently as mild-to-moderate headache lasting < 1 h. Enquiry timeframes were 1 year, 4 weeks and 1 day (headache yesterday [HY]).

**Results:**

Of 2,790 participants, two thirds (68.5%) reported headache in the preceding year (age- and gender-adjusted 1-year prevalence: 66.3%). HY was reported by 20.4%, a proportion that might be expected to have headache on any day. Headache on ≥ 15 days/month (H15+) was common (4.1%), with probable medication-overuse headache (pMOH) accounting for 0.5% (0.2% among children, 0.7% among adolescents; *p* = 0.06). UdH took diagnostic precedence over migraine and tension-type headache (TTH), so all other estimates were influenced by the definition of UdH. By the conventional (initial) definition, 1-year prevalence of UdH was 18.2%; by the modified definition, it was 30.2%. Estimates for migraine and TTH, 29.1% and 13.1% respectively with UdH defined conventionally, were reduced to 22.2% and 9.5% with the modified definition.

**Conclusions:**

Headache is very common among children and adolescents in Georgia. The findings raise particular concern about the prevalence of H15 + in young people, with a strong trend of increasing pMOH with age. They also highlight the diagnostic uncertainties inherent in epidemiological studies in these age groups. With the definition of UdH a dominating issue, the findings support the view that it needs to be modified, without offering a definitive solution.

## Background

Against a background of still relatively sparse knowledge, the Global Campaign against Headache [1, 2] is undertaking a worldwide programme of schools-based studies of headache prevalence and attributed burden among children (aged 6–11 years) and adolescents (aged 12–17 years) [2]. The purpose is to expand our understanding not only of headache prevalence but also of its characteristics and their consequences for health and (future) wellbeing among these age groups. This understanding may support interventions offering both short- and long-term benefits.

The programme uses a data-acquisition technique that cluster-samples the world, performing schools-based cross-sectional surveys in countries from all World Regions. It focuses on the primary headache disorders that are of public-health importance: migraine and tension-type headache (TTH), together with undifferentiated headache (UdH), a common disorder among these age groups, which may later, with brain maturation, evolve into migraine or TTH [3]. The programme also includes medication-overuse headache (MOH) and other headache on ≥ 15 days/month (other H15+), since these, also, have major impacts on population health even among these young age groups.

This study in Georgia was the fourth in the European Region, following similar studies in Turkey [3, 4], Austria [5] and Lithuania [6]. Others have so far been conducted in African [7–9], Eastern Mediterranean [10], South-East Asia [11] and Western Pacific Regions [12]. Georgia, an upper-middle income [13] but developing country in Eastern Europe, has a population of about 3.8 million [14], of whom about 8.9% are aged 6–17 years [15]. Education is free, and compulsory from the age of 6 until 17–18 years, so schools-based sampling is an appropriate means of gathering a sample representative of the population of interest. Urban/rural divide is approximately 62:38 [14].

The aim of the study was to establish the prevalence of headache overall and of each headache type, and their demographic associations. The purposes of the study were two-fold: to add to understanding of the global burden of headache, and, importantly, to inform health and educational policies in Georgia. A later manuscript will describe and quantify the components of ill-health burden attributed to each headache type.

## Methodology

We conducted a cross-sectional survey in schools across Georgia following the programme’s standardised protocol [4]. While the study was intended to be nationally representative, we were unable to include the regions of Abkhazia and Tskhinvali (South Ossetia), both occupied by Russia.

Pupils completed a structured questionnaire in class, under supervision.

### Ethics and approvals

The protocol was approved by David Tvildiani Medical University Ethics Committee (N4, 23.02.2024).

Permission to perform this study was granted by the Georgian Ministry of Education. Additionally, permissions were obtained from academic and administrative authorities of each school, along with the consents of principals and class teachers.

All pupils participating in the study were informed of its nature and purpose, and gave oral consent before enrolment. Continuing consent was evidenced by completion of the questionnaires. Since the study was wholly innocuous, the ethics committee agreed that prior parental consent was not additionally needed.

Data were recorded anonymously, and managed in accordance with local data protection regulations.

### Sampling and recruitment

Data collection took place between December 2022 and November 2023.

Excepting the inaccessible areas (see above), we aimed for a sample matching the urban/rural divide of Georgia (62:38 [14]). To achieve this, we included a total of nine schools in regions distributed from west to east of the country: five in urban centres (four in Tbilisi, the capital city, towards the east; one in Batumi, on the Black Sea [western] coast) and one in each of four rural regions (Chakvi in the west, Tserovani towards the north, Borjomi in the south, and Telavi in the east).

We aimed for > 2,100 participants overall in accordance with published consensus guidelines, which assert that the gain from sample sizes of > 2,000 is small and often not worthwhile [16]. Within this total, we aimed to recruit about 50% from Tbilisi, 10% from Batumi and 13% from each of the other three regions.

Eligibility criteria were age 6–11 years (children) or 12–17 years (adolescents), and presence at the school on the day of survey. Pupils who were unwilling to participate, or for any reason could not, were counted as non-participants; those absent on the day were not, since they were not available for inclusion.

### Data collection

We used the child and adolescent versions of the Headache-Attributed Restriction, Disability, Social Handicap and Impaired Participation (HARDSHIP) questionnaire [4]. These were translated into Georgian language following the Global Campaign’s translation guidelines for lay documents [17]. A pilot study in 200 children and 300 adolescents confirmed usability and comprehensibility in these age groups.

The questionnaires began with demographic enquiry, which was followed by neutral headache screening questions (“have you ever had headache?”, and “have you had headache in the last year?”). For those responding positively to the latter, diagnostic questions were followed by enquiries into attributable burden (to be reported later). The timeframe of these enquiries was the preceding 4 weeks (28 days), with additional questions about headache on the day before the survey (headache yesterday [HY]) included to identify and circumvent recall error [16].

These questionnaires were completed by the pupils in class, under supervision by the researchers and class teacher, with guidance and clarification provided when needed. Separate questionnaires, completed by the teachers, enquired into each school’s location (urban or rural), and the estimated proportions of pupils coming from low-income homes or travelling for > 1 h each day.

### Headache diagnoses

Diagnostic questions were based on modified ICHD-3 criteria [18], except those for UdH. Diagnoses were made by applying the HARDSHIP algorithm to pupils’ responses to these questions [4, 19]. The algorithm first identified pupils reporting headache on ≥ 14 days in the previous 28 days, which was assumed to equate to ≥ 15 days in the previous 30 days (H15+). Those also reporting acute medication use on ≥ 14 days were diagnosed as probable MOH (pMOH); the others were not further diagnosed (other H15+). Among the remainder (those reporting headache on < 14 days in the previous 28), diagnostic precedence was given to UdH, followed, in order, by definite migraine, definite TTH, probable migraine and probable TTH [4]. UdH was initially defined conventionally as headache of mild intensity and usual duration of < 1 h [3], but subsequently (modified definition) as mild or moderate headache lasting < 1 h (see Results and Discussion).

In subsequent analyses, definite and probable migraine were combined, as were definite and probable TTH.

### Data management

Questionnaires were securely stored at David Tvildiani Medical University. Data were entered into SPSS. To ensure accuracy, independent double data entry was performed, and discrepancies reconciled by cross-referencing to the original data.

### Analysis

The principal analysis was performed at University of Mersin, using the analytical methods adopted by all previous Global Campaign studies [3–12]. Additional statistical modelling, specifically to review and modify the diagnostic criteria for UdH, was performed at Norwegian Centre for Headache Research (NorHead).

We categorised schools as urban or rural, and assumed all pupils in those schools were, respectively, urban or rural dwellers. As a socio-economic indicator, we categorised schools by teachers’ estimates of proportions of pupils coming from low-income homes (< 25%, 25–50%, > 50% [for simplicity we refer to these categories as “high-income”, “middle-income” and “low-income” schools]). In accordance with the standardised protocol [4], we also characterised schools by proportions of pupils living at distances of > 1 h’s travel time.

For descriptive statistics, we used means and standard deviations (SDs) for continuous variables, and proportions (%) with 95% confidence intervals (CIs) for categorical variables.

We calculated observed prevalence of each headache type in the sample, and made population estimates by adjusting for gender and age using data from the United Nations Department of Economic and Social Affairs [20]. To examine relationships between headache types and gender, age, school income category and school location (urban or rural), we performed both bivariate analyses, reporting odds ratios (ORs), and logistic regression, reporting adjusted ORs (aORs).

We calculated 1-day prevalence of any headache as the proportion of the sample reporting HY. We also calculated the proportion reporting HY of those with any headache in the preceding year, along with proportions predicted to have HY from the responses to two questions: “On how many days in the last week did you have headache?” and “On how many days in the past four weeks did you have headache?”

The significance level for all analyses was set at *p* < 0.05.

## Results

The surveyed sample included 2,790 pupils from the nine schools. Class registers indicated a total of 3,586 enrolled pupils (male 1,780 [49.6%]; female 1,806 [50.4%]), but 788 (mostly adolescents) were not present on the survey days. Six pupils did not participate, and two were excluded because of incomplete diagnostic data. Therefore, the non-participating proportion was 8/(3,586–788) = 0.3%.

Table 1 shows demographic and school variables. There were almost equal proportions of males (48.9%) and females (51.1%), and of children (50.6%) and adolescents (49.4%). There were 1,347 (48.3%) pupils from the five urban schools and 1,443 (51.7%) from the four rural schools, a mismatch of the national urban/rural divide (62:38) [14]. Most pupils (69.9%) were from schools categorized as “high-income” (although it should be recognized that this was a relative term). Numbers travelling long distances to school were estimated to be low (Table 1).

### Observed prevalence

A total of 1,910 (843 males, 1,067 females; 755 children, 1,155 adolescents) reported headache of any type in the preceding year.

In the following tables we report proportions diagnosed with each headache type according to the two definitions of UdH. Since pMOH (0.5%; males 0.3%, females 0.6%) and other H15+ (3.7%; males 2.1%, females 5.3%) took diagnostic precedence, proportions for these did not vary (Tables 2A, 2B).

With the conventional definition, 19.0% met criteria for UdH, with no significant gender difference (Table 2A). With this definition, those with headache duration < 1 h and sufficient other characteristics of migraine, including moderate headache, met ICHD criteria for probable migraine (23.9% overall; males 18.7%, females 28.9%) (Table 2). With the modified criteria (so that those with headache duration < 1 h and moderate headache would be classified as UdH regardless of other symptoms), the proportion diagnosed as UdH inevitably increased, to 31.3%, still with no significant gender difference, while the estimates for probable migraine fell commensurately to 16.6% overall, 13.2% among males and 19.9% among females. Proportions diagnosed with definite migraine remained unchanged (Table 2B).

**Table 1:** Demographic and school variables

Variable of interest	Schools (*N* = 9) *n*	Pupils (*N* = 2,790) *n* (%)
Gender		
Male		1,364 (48.9)
Female		1,426 (51.1)
Age (years)		
6–11		1,411 (50.6)
12–17		1,379 (49.4)
Habitation		
Urban	5	1,347 (48.3)
Rural	4	1,443 (51.7)
Proportion of pupils from low-income homes		
< 25% (“high-income school”)	7	1,952 (69.9)
25–50% (“middle-income school”)	2	838 (30.1)
> 50% (“low-income school”)	0	0
Proportion of pupils travelling > 1 h		
< 25%	7	2,060 (73.8)
25–50%	2	730 (26.2)
> 50%	0	0

Proportions diagnosed with TTH fell from 13.4% overall (Table 2A) to 9.7% (Table 3B), with slight male preponderance in each case expressed in definite TTH. Unclassified headaches were 1.7% falling to 0.3% (Tables 2A, 2B).

**Table 2:** **A**. Observed 1-year prevalence by headache type and gender, according to conventional criteria for undifferentiated headache (mild headache lasting < 1 h). **B**. Observed 1-year prevalence by headache type and gender, according to modified criteria for undifferentiated headache (mild or moderate headache lasting <1 hour)

Headache type	Overall	Male	Female
	% [95% confidence interval]		
Any headache	68.5 [66.7–70.2]	61.8 [59.2–64.4]	74.8 [72.6–77.1]
Migraine	30.2 [28.5–31.9]	24.3 [22.0-26.5]	35.9 [33.4–38.4]
definite	6.3 [5.4–7.2]	5.6 [4.4–6.8]	7.0 [5.7–8.3]
probable	23.9 [22.3–25.5]	18.7 [16.6–20.8]	28.9 [26.5–31.2]
TTH	13.4 [12.1–14.6]	14.8 [12.9–16.7]	12.0 [10.3–13.7]
definite	6.5 [5.6–7.4]	7.9 [6.5–9.4]	5.1 [4.0-6.3]
probable	6.9 [5.9–7.8]	6.9 [5.5–8.2]	6.9 [5.6–8.2]
pMOH	0.5 [0.2–0.7]	0.3 [0.0-0.6]	0.6 [0.2-1.0]
Other H15+	3.7 [3.0-4.4]	2.1 [1.3–2.8]	5.3 [4.2–6.5]
UdH	19.0 [17.5–20.4]	18.5 [16.4–20.5]	19.4 [17.4–21.5]
Unclassified	1.7 [1.2–2.2]	1.9 [1.2–2.6]	1.5 [9.0-2.2]

Headache type	Overall	Male	Female
	% [95% confidence interval]		
Any headache	68.5 [66.7–70.2]	61.8 [59.2–64.4]	74.8 [72.6–77.1]
Migraine	22.9 [21.4–24.5]	18.8 [16.7–20.8]	26.9 [24.6–29.2]
definite	6.3 [5.4–7.2]	5.6 [4.4–6.8]	7.0 [5.7–8.3]
probable	16.6 [15.2–18.0]	13.2 [11.4–15.0]	19.9 [17.8–22.0]
TTH	9.7 [8.6–10.8]	10.6 [9.0-12.3]	8.9 [7.4–10.4]
definite	6.5 [5.6–7.4]	7.9 [6.5–9.4]	5.1 [4.0-6.3]
probable	3.3 [2.6–3.9]	2.7 [1.9–3.6]	3.8 [2.8–4.8]
pMOH	0.5 [0.2–0.7]	0.3 [0.0-0.6]	0.6 [0.2-1.0]
Other H15+	3.7 [3.0-4.4]	2.1 [1.3–2.8]	5.3 [4.2–6.5]
UdH	31.3 [29.6–33.0]	29.8 [27.3–32.2]	32.8 [30.4–35.3]
Unclassified	0.3 [0.1–0.4]	0.3 [0.0-0.6]	0.2 [0.0-0.4]

*TTH* Tension type headache, *pMOH,* Probable medication overuse headache, *H15+* Headache on ≥15 days/month,* UdH* Undifferentiated headache

**Table 3:** **A**. One-year prevalence estimates by headache type and demographic variable according to conventional criteria for undifferentiated headache (mild headache lasting < 1 h). **B**. One-year prevalence estimates by headache type and demographic variable according to modified criteria for undifferentiated headache (mild or moderate headache lasting <1 hour)

Variable	Any headache	Migraine	TTH	pMOH	Other H15+	UdH
	% [95% confidence interval]					
Observed						
Overall	68.5 [66.7–70.2]	30.2 [28.5–31.9]	13.4 [12.1–14.6]	0.5 [0.2–0.7]	3.7 [3.0-4.4]	19.0 [17.5–20.4]
Gender						
Male	61.8 [59.2–64.4]	24.3 [22.0-26.5]	14.8 [12.9–16.7]	0.3 [0.0-0.6]	2.1 [1.3–2.8]	18.5 [16.4–20.5]
Female	74.8 [72.6–77.1]	35.9 [33.4–38.4]	12.0 [10.3–13.7]	0.6 [0.2-1.0]	5.3 [4.2–6.5]	19.4 [17.4–21.5]
Age (years)						
6–11	54.5 [50.9–56.1]	22.0 [19.9–24.2]	10.1 [8.6–11.7]	0.2 [0.0-0.5]	1.5 [0.9–2.1]	17.4 [15.4–19.3]
12–17	83.8 [81.8–85.7]	38.6 [36.0-41.1]	16.7 [14.7–18.6]	0.7 [0.3–1.2]	6.0 [4.8–7.3]	20.6 [18.5–22.7]
School income category						
“high-income”	71.1 [69.0-73.1]	32.8 [30.8–34.9]	13.7 [12.2–15.3]	0.5 [0.2–0.8]	4.3 [3.4–5.1]	18.0 [16.3–19.7]
“middle-income”	62.4 [59.1–65.7]	24.1 [21.2–27.0]	12.5 [10.3–14.8]	0.4 [0.0-0.8]	2.5 [1.4–3.6]	21.2 [18.5–24.0]
School location						
Urban	71.3 [68.9–73.8]	33.0 [30.5–35.5]	13.0 [11.2–14.8]	0.5 [0.1–0.9]	4.8 [3.6–5.9]	18.4 [16.3–20.5]
Rural	65.8 [63.3–68.2]	27.7 [25.3–30.0]	13.7 [11.9–15.5]	0.4 [0.1–0.7]	2.8 [1.9–3.6]	19.5 [17.4–21.5]

Variable	Any headache	Migraine	TTH	pMOH	Other H15+	UdH
	% [95% confidence interval]					
Observed						
Overall	68.5 [66.7–70.2]	22.9 [21.4–24.5]	9.7 [8.6–10.8]	0.5 [0.2–0.7]	3.7 [3.0-4.4]	31.3 [29.6–33.0]
Gender						
Male	61.8 [59.2–64.4]	18.8 [16.7–20.8]	10.6 [9.0-12.3]	0.3 [0.0-0.6]	2.1 [1.3–2.8]	29.8 [27.3–32.2]
Female	74.8 [72.6–77.1]	26.9 [24.6–29.2]	8.9 [7.4–10.4]	0.6 [0.2-1.0]	5.3 [4.2–6.5]	32.8 [30.4–35.3]
Age (years)						
6–11	54.5 [50.9–56.1]	15.8 [13.9–17.7]	6.2 [5.0-7.5]	0.2 [0.0-0.5]	1.5 [0.9–2.1]	29.4 [27.0-31.8]
12–17	83.8 [81.8–85.7]	30.2 [27.8–32.7]	13.3 [11.5–15.1]	0.7 [0.3–1.2]	6.0 [4.8–7.3]	33.2 [30.8–35.8]
School income category						
“high-income”	71.1 [69.0-73.1]	25.6 [23.6–27.5]	10.0 [8.7–11.4]	0.5 [0.2–0.8]	4.3 [3.4–5.1]	30.3 [28.3–32.4]
“middle-income”	62.4 [59.1–65.7]	16.8 [14.3–19.4]	9.1 [7.1–11.0]	0.4 [0.0-0.8]	2.5 [1.4–3.6]	33.7 [30.5–36.9]
School location						
Urban	71.3 [68.9–73.8]	26.4 [24.1–28.8]	10.0 [8.4–11.6]	0.5 [0.1–0.9]	4.8 [3.6–5.9]	29.4 [27.0-31.8]
Rural	65.8 [63.3–68.2]	19.7 [17.6–21.7]	9.5 [8.0–11.0]	0.4 [0.1–0.7]	2.8 [1.9–3.6]	33.1 [30.7–35.6]

*TTH* Tension type headache, *pMOH* Probable medication overuse headache, *H15+* Headache on ≥15 days/month, *UdH* Undifferentiated headache

**Table 4:** **A**. Unadjusted analyses of associations (bivariate analysis) between headache types and demographic variables according to conventional criteria for undifferentiated headache (mild headache lasting < 1 h). **B**. Unadjusted analyses of associations (bivariate analysis) between headache types and demographic variables according to modified criteria for undifferentiated headache (mild or moderate headache lasting <1 hour)

Variable	Migraine	TTH	pMOH	Other H15+	UdH
	Odds ratio [95% confidence interval]; *p*				
Gender					
Male	reference	reference	reference	reference	reference
Female	**1.7 [1.4–2.1]** ***p*** **< 0.001**	**0.8 [0.6-1.0]** ***p*** **= 0.03**	2.2 [0.7-8.0] *p* = 0.20	**2.7 [1.8–4.2]** ***p*** **< 0.001**	1.1 [0.9–1.3] *p* = 0.52
Age (years)					
6–11	reference	reference	reference	reference	reference
12–17	**2.2 [1.9–2.6]** ***p*** **< 0.001**	**1.8 [1.4–2.2] ** ***p*** **< 0.001**	3.4 [1.0-15.3] *p* = 0.06	**4.2 [2.7–7.1]** ***p*** **< 0.001**	**1.2 [1.0-1.5]** ***p*** **= 0.03**
School income category					
“high-income”	reference	reference	reference	reference	reference
“middle-income”	**0.6 [0.5–0.8]** ***p*** **< 0.001**	0.9 [0.7–1.1] *p* = 0.39	0.7 [0.2–2.3] *p* = 0.59	**0.6 [0.3–0.9]** *p* **= 0.03**	**1.2 [1.0-1.5]** *p* **= 0.04**
School location					
Urban	reference	reference	reference	reference	reference
Rural	**0.8 [0.7–0.9]** *p* **= 0.002**	1.1 [0.9–1.3] *p* = 0.57	0.8 [0.3–2.4] *p* = 0.69	**0.6 [0.4–0.9]** *p* **= 0.006**	1.1 [0.9–1.3] *p* = 0.48

Variable	Migraine	TTH	pMOH	Other H15+	UdH
	Odds ratio [95% confidence interval]; *p*				
Gender					
Male	reference	reference	reference	reference	reference
Female	**1.6 [1.3–1.9]** *p* **< 0.001**	0.8 [0.6–1.1] *p* = 0.13	2.2 [0.7-8.0] *p* = 0.20	**2.7 [1.8–4.2]** *p* **< 0.001**	1.2 [1.0-1.4] *p* = 0.08
Age (years)					
6–11	reference	reference	reference	reference	reference
12–17	**2.3 [1.9–2.8]** *p* **< 0.001**	**2.3 [1.8-3.0]** *p* **< 0.001**	3.4 [1.0-15.3] *p* = 0.06	**4.2 [2.7–7.1]** *p* **< 0.001**	**1.2 [1.0-1.4]** *p* **= 0.03**
School income category					
“high-income”	reference	reference	reference	reference	reference
“middle-income”	**0.6 [0.5–0.7]** *p* **< 0.001**	0.9 [0.7–1.2] *p* = 0.43	0.7 [0.2–2.3] *p* = 0.59	**0.6 [0.3–0.9]** *p* **= 0.03**	1.2 [1.0-1.4] *p* = 0.08
School location					
Urban	reference	reference	reference	reference	reference
Rural	**0.7 [0.6–0.8]** *p* **< 0.001**	0.9 [0.7–1.2] *p* = 0.64	0.8 [0.3–2.4] *p* = 0.69	**0.6 [0.4–0.9]** *p* **= 0.006**	**1.2 [1.0-1.4]** *p* **= 0.03**

*TTH* Tension type headache, *pMOH* Probable medication overuse headache, *H15+* Headache on ≥15 days/month, *UdH* Undifferentiated headache, significant p-values (<0.05) are emboldened

**Table 5:** **A**. Adjusted analyses of associations (logistic regression) between headache types and demographic variables according to conventional criteria for undifferentiated headache (mild headache lasting < 1 h). **B**. Adjusted analyses of associations (logistic regression) between headache types and demographic variables according to modified criteria for undifferentiated headache (mild or moderate headache lasting <1 hour)

Variable	Migraine	TTH	pMOH	Other H15+	UdH	
	Odds ratio [95% confidence interval]; *p*					
Gender						
Male	reference	reference	reference	reference	reference	
Female	**1.7 [1.5–2.1]** *p* **< 0.001**	**0.8 [0.6-1.0]** *p* **= 0.02**	2.1 [0.7–7.8] *p* = 0.22	**2.7 [1.7–4.2]** *p* **< 0.001**	1.1 [0.9–1.3] *p* = 0.48	
Age (years)						
6–11	reference	reference	reference	reference	reference	
12–17	**2.3 [1.9–2.7]** *p* **< 0.001**	**1.8 [1.4–2.2]** *p* **< 0.001**	3.4 [1.0-15.2] *p* = 0.06	**4.3 [2.7–7.1]** *p* **< 0.001**	**1.2 [1.0-1.5]** *p* **= 0.03**	
School income category						
“high-income”	reference	reference	reference	reference	reference	
“middle-income”	**0.7 [0.5–0.9]** *p* **= 0.001**	0.8 [0.6-1.0] *p* = 0.09	0.8 [0.1–4.2] *p* = 0.74	0.9 [0.5–1.6] *p* = 0.63	**1.3 [1.0-1.7]***p***= 0.04**	
School location						
Urban	reference	reference	reference	reference	reference	
Rural	1.0 [0.8–1.2] *p* = 0.66	1.2 [0.9–1.6] *p* = 0.14	0.9 [0.2–3.3] *p* = 0.90	0.6 [0.3-1.0] *p* = 0.06	0.9 [0.7–1.2] *p* = 0.44	

Variable	Migraine	TTH	pMOH	Other H15+		UdH
	Odds ratio [95% confidence interval]; *p*					
Gender						
Male	reference	reference	reference	reference		reference
Female	**1.6 [1.3–1.9]** *p* **< 0.001**	0.8 [0.6-1.0] *p* = 0.09	2.1 [0.7–7.8] *p* = 0.22	**2.7 [1.7–4.2]** *p* **< 0.001**		1.2 [1.0-1.4] *p* = 0.08
Age (years)						
6–11	reference	reference	reference	reference		reference
12–17	**2.3 [1.9–2.8]** *p* **< 0.001**	**2.3 [1.8-3.0]** *p* **< 0.001**	3.4 [1.0-15.2] *p* = 0.06	**4.3 [2.7–7.1]** *p* **< 0.001**		**1.2 [1.0-1.4]** *p* **= 0.03**
School income category						
“high-income”	reference	reference	reference	reference		reference
“middle-income”	**0.7 [0.5–0.9]** *p* **= 0.003**	0.9 [0.6–1.3] *p* = 0.46	0.8 [0.1–4.2] *p* = 0.74	0.9 [0.5–1.6] *p* = 0.63		1.1 [0.9–1.3] *p* = 0.56
School location						
Urban	reference	reference	reference	reference		reference
Rural	0.8 [0.7-1.0] *p* = 0.12	1.0 [0.7–1.4] *p* = 0.95	0.9 [0.2–3.3] *p* = 0.90	0.6 [0.3-1.0] *p* = 0.06		1.1 [0.9–1.4] *p* = 0.20

*TTH* Tension type headache, *pMOH* Probable medication overuse headache, *H15+* Headache on ≥15 days/month, *UdH* Undifferentiated headache, significant p-values (<0.05) are emboldened

**Table 6:** Age- and gender-adjusted one-year prevalence estimates for all headache and each headache type according to the two definitions of undifferentiated headache

UdH definition	Any headache	Migraine	TTH	pMOH	Other H15+	UdH
	% [95% confidence interval]					
Conventional						
(mild headache lasting < 1 h)	66.3[64.5–68.0]	29.1[27.5–30.9]	13.1[11.9–14.4]	0.5[0.3–0.8]	3.6[2.9–4.4]	18.2[16.8–19.7]
Modified						
(mild or moderate headache lasting < 1 h)	66.3[64.5–68.0]	22.2[20.7–23.8]	9.5[8.5–10.7]	0.5[0.3–0.8]	3.6[2.9–4.4]	30.2[28.6–32.0]

*UdH* Undifferentiated headache, *TTH* Tension type headache, *pMOH* Probable medication overuse headache, *H15+* Headache on ≥15 days/month

**Table 7:** Reported and predicted headache yesterday, overall and by gender and age

	Recalled mean headache frequency		Headache yesterday (proportion of those with any headache in the preceding year)		
	Days in preceding 1 week (F7)	Days in preceding 4 weeks (F28)	Reported *n* (%)	Predicted (%)	
				from 1-week recall (F7*100/7)^a^	from 4-week recall (F28*100/28)^a^
Any headache (*n* = 1,910)	1.5	4.3	568 (29.7)	22.0	15.3
males (*n* = 843)	1.2	3.4	162 (19.2)	17.7	12.1
females (*n* = 1,067)	1.8	5.0	406 (38.1)	25.5	17.7
children (*n* = 755)	1.4	3.5	181 (24.0)	20.2	12.4
adolescents (*n* = 1,155)	1.6	4.8	387 (33.5)	23.3	17.2

^a^Apparent discrepancies are due to rounding of the values shown for F7 and F28

### Associations

These are set out in Tables 3A, 3B, 4A, 4B, 5A and 5B. There were the usual associations with gender (prevalences of any headache and of each type other than TTH higher among females, although not in all cases significantly) and age (all prevalences higher among adolescents, again not in all cases significantly) (Tables 3A, 3B). These associations were unchanged by modification of UdH definition. Although UdH was more prevalent among adolescents than children, it declined as a proportion of all headache from 31.9 to 24.6% (conventional definition; Table 3A) or from 53.9 to 39.6% (modified definition; Table 3B).

Associations are more explicitly illustrated in Tables 4A and 4B (bivariate analysis) and in Tables 5A and 5B (logistic regression). All headache types were positively associated with age (pMOH, with small numbers, on the verge of significance). Migraine and other H15 + were associated with female gender. Migraine was positively associated with high-income school category (Tables 4A, 4B, 5A, 5B). Its apparent association with urban school location (Tables 4A, 4B) disappeared in logistic regression (Tables 5A, 5B).

### Age- and gender-adjusted prevalence estimates

In view of these associations, we adjusted prevalence estimates for age and gender (Table 6). Adjusted estimates were not greatly different from observed proportions (Table 3A, 3B). One-year prevalence of any headache was 66.3%, of pMOH 0.5% and of other H15 + 3.6%. Estimates for UdH were 18.2% by the conventional definition and 30.2% by the modified definition. Corresponding estimates for migraine were 29.1% and 22.2%, and for TTH 13.1% and 9.5%.

### Headache yesterday

Among all pupils (*N* = 2,790), 568 reported HY, a 1-day prevalence of any headache of 20.4% [18.9–21.9] (i.e., one fifth of children and adolescents might be expected to have headache on any day).

Of pupils reporting headache in the past year (*n* = 1,910), 29.7% reported HY, twice as many females (38.1%) as males (19.2%) and more adolescents (33.5%) than children (24.0%) (Table 7). These numbers exceeded predictions based on recalled frequencies, by a factor of about 2 with 4-week recall, by a smaller factor (< 1.5) with 1-week recall.

## Discussion

In this schools-based survey of children and adolescents in Georgia, using well-tried methodology [4–12], we found high proportions with headache. More than two thirds (68.5%) of our sample of *N* = 2,790 reported headache of any type in the preceding year, yielding an age- and gender-adjusted 1-year prevalence estimate of 66.3%. One fifth (20.4%) reported HY, a proportion, as noted, that might be expected to have headache on any day. H15 + was common: estimated prevalence was 4.1%, with pMOH accounting for a small proportion (0.5%). Since we gave diagnostic precedence to UdH over migraine and TTH, all other estimates were influenced by the definition of UdH. By the conventional definition (mild headache, duration < 1 h), 1-year prevalence of UdH was 18.2%; by the modified definition (mild or moderate headache, duration < 1 h), it was a much higher 30.2%. Estimates for migraine and TTH, 29.1% and 13.1% respectively with UdH defined conventionally, were reduced to 22.2% and 9.5% with the modified definition.

The definition of UdH is therefore the dominating issue here. It has arisen before, notably in our similar study in Nepal [11]. In that study, we commented on the uncertainties of diagnosis reliant upon subjective reporting by young people of symptoms elicited by questionnaire, the only means available in large cross-sectional epidemiological studies. These uncertainties are compounded by the questionable applicability of ICHD criteria [18], based on adult symptomatology, to children and adolescents [3, 21]. It is clear that young people commonly have headache that expresses differently from migraine or TTH in adults [3–12]. It may evolve into one or other – we need longitudinal studies to determine this. The most prominent distinction is short duration [3], with large proportions reporting headache with typical duration of < 1 h. We strongly believe that broadening the definition of migraine to fit young people within its scope [21] is the wrong approach. The evolving nature of headache expressed by the maturing brain should be recognised, and diagnostic criteria adapted with this in mind: hence the concept of UdH [3]. However, the problem of defining UdH has no resolution with our current state of knowledge, although we believe its distinction from migraine or TTH ought not to turn upon the distinction between mild and moderate headache, which is highly subjective at any age [11]. Short duration is the essential criterion.

When we apply the modified criteria for UdH, this headache type becomes the most common among both children and adolescents, although UdH declines as a proportion of all headache among adolescents, while migraine in this age group becomes almost as common (30.2% *versus* 33.2%), with prevalence double that among children (15.8%). TTH also doubles in prevalence among adolescents compared with children (13.3% *versus* 6.2%). These associations appear intuitively correct, if, as we have argued [3], UdH is a headache type evolving, with ageing, into adult types.

In summary, we believe there are convincing arguments for modifying the criteria for UdH. Here and earlier [11], we have proposed inclusion of moderate headache of duration < 1 h, but believe that definitive resolution of this question is not yet possible.

We noted the usual associations with gender (prevalences of all headache types other than TTH were higher among females) and age (all prevalences were higher among adolescents), although not all were significant. Importantly, these associations were not changed by modifying the definition of UdH. In logistic regression analysis, migraine was positively associated with female gender (aOR 1.6) and adolescence (aOR 2.3), both as expected.

In both bivariate and logistic regression analyses, migraine was positively associated with school income category, regardless of UdH definition (aOR 0.6 and 0.7 for middle-income *versus* high-income as reference). This should be interpreted very cautiously. The assessment of income level was inexact, relying on teachers’ judgements of how many pupils came from low-income homes. This form of enquiry was developed with low and lower-middle income countries in mind, where variations in income might be expected to have impact, whereas in Georgia, an upper-middle income country [13], no schools were assessed as ”low-income”. A similar association in Lithuania lost significance in logistic regression [6]. Differences were insignificant in Austria [5] and Iran [10], while in Mongolia the association was reversed [12].

Other H15 + was positively associated with female gender (aOR 2.7) and adolescence (aOR 4.3). pMOH showed an increasing trend with age (aOR 3.4; *p* = 0.06), which should be of public-health concern.

Data exist for adults in Georgia, from a population-based survey conducted by door-to-door enquiry in 2008 [22, 23]. Observed 1-year prevalence of migraine was 15.6%, of TTH 37.3% and of H15 + 7.6%. The last included an uncertain 0.9% with pMOH [23]. These data may suggest that what presents as migraine in young people loses the characteristics of this disorder for many with progression into adulthood. However, there are multiple caveats. The adult study, one of the earliest conducted within the Global Campaign against Headache, did not use the HARDSHIP questionnaire [22]. Enquiry into headache was prejudiced by a screening question focused on headache “not related to flu, hangover, cold or head injury” [22], since recognised as likely to introduce bias [16]. Of those reporting headache, 19% were deemed unclassifiable because of incomplete responses [23].

We have data from children and adolescents from Lithuania [6]. Like Georgia, Lithuania is a post-Soviet country, although the latter is now within the European Union and more economically advanced, and the two countries are not ethnically or otherwise culturally related. The study in Lithuania used identical methodology to the present one [6, 24]. Gender- and age-adjusted 1-year prevalence estimates were 21.4% for migraine, 25.6% for TTH, 24.0% for UdH (conventional definition), 0.8% for pMOH and 3.1% for other H15+ [6]. HY was reported by 17.5%, which, as in Georgia, was almost double the 9.8% predicted from 1-year prevalence and recalled frequency (over 4 weeks) [6]. These estimates differ somewhat from those in Georgia, but age- and gender-adjusted estimates after modification of UdH definition are not available from Lithuania.

We also have data from neighbouring Turkey, collected in 2014, again using identical methodology [4]. Georgia and Turkey are also ethnically unrelated (Georgians being an autochthonous people), and culturally distinct. The data from Turkey have not been fully reported, but estimated 1-year prevalence of UdH was 29.2% (conventional definition), of migraine 26.7%, of TTH 12.9%, of pMOH 0.9%, of other H15 + 3.4% [3].

What is common to all of these studies in children and adolescents is the high proportion with H15+: 4.1% in Georgia (pMOH 0.5%), 3.9% in Lithuania (pMOH 0.8%) [6], 4.3% in Turkey (pMOH 0.9%) [3]. The nature of other H15 + is not known, but it is a risk factor for MOH [25]. In Georgia and Lithuania [6], the prevalence of pMOH advanced with age.

H15 + is obviously an important contributor to HY. Of pupils in Georgia reporting headache in the past year, almost one quarter (24.0%) of children and one third (33.5%) of adolescents reported headache on the day prior to the survey. These proportions exceeded predictions based on recalled headache frequencies over the preceding week or 4 weeks, by factors of 1.5 to 2, an indication that recall of frequency is unreliable (significantly underestimating actual frequency). Since similar findings have come from other studies [6–12], this is of importance not only epidemiologically but also clinically.

The particular strengths of this study were its use of standardised and well-tested methodology [4–12], a high N (2,790) and a very high participating proportion (99.7%). The principal limitation – the uncertain reliability of information gathered from children – has been discussed, along with the underlying limitation that the ICHD criteria do not apply well to children [21]. While it is necessary to include UdH as a separate diagnosis in order to give a full account of headache among children and adolescents, it is not yet established how UdH should be defined. Because of the considerable challenges to doing so in young people, we did not undertake psychometric assessment of the Georgian versions of the HARDSHIP questionnaires, or directly test their reliability in the target populations. The questionnaires themselves have been used in nine previous studies and eleven translations [3–12]. As noted earlier, the study was representative only of those parts of the country that were accessible to us, which did not include the occupied regions of Abkhazia and Tskhinvali (South Ossetia). Despite that schooling is compulsory, 788 (22.0%) of 3,586 enrolled pupils were not present on the survey days, a level of absenteeism, particularly among adolescents, not uncommon in Georgia. Specific reasons were indeterminable, but this was a possible source of bias. Since HY was reported by 20.4% of participants, this was the likely proportion with headache on each survey day. To the extent this led to absenteeism, excluding a group with headache from the prevalence count, it would have been a cause of underestimation. We did not adjust prevalence estimates for the sample imbalance in urban/rural divide, but classification was of schools, not pupils’ dwellings, and, further, apparent associations with urban dwelling (which might have led to under- rather than overestimation) disappeared in logistic regression.

## Conclusion

These findings show that headache is very common among children and adolescents in Georgia. They raise concern, particularly with regard to the high prevalence of H15 + in young people, with a strong trend of increasing pMOH with advancing age. They also highlight the diagnostic uncertainties inherent in epidemiological studies in these age groups, and support our earlier-expressed view [11] that the definition of UdH, which was proposed in 2018 [3], needs modification.

## Data Availability

The original data are held at David Tvildiani Medical University, and the analytical set at University of Mersin and at Norwegian University of Science and Technology. When analyses are completed, anonymised data will be available on request for academic purposes, in line with the policy of the Global Campaign against Headache.

## Abbreviations

AOR: Adjusted Odds Ratio
CI: Confidence Interval
d/m: Days/month
DTMU: David Tvildiani Medical University
GBD: : Global Burden of Disease (Study)
H15+: Headache on ≥15 days/month
HARDSHIP: Headache-Attributed Restriction, Disability, Social Handicap and Impaired Participation (Questionnaire)
HY: Headache yesterday
ICHD: International Classification of Headache Disorders
LTB: *Lifting The Burden*
MOH: Medication-overuse headache
Norhead: Norwegian Centre for Headache Research
NTNU: Norwegian University of Science and Technology
OR: Odds ratio
Pmoh: Probable MOH
SD: Standard deviation
TTH: Tension-type headache
UDH: Undifferentiated headache
